# SHR-A1811, a novel anti-HER2 antibody–drug conjugate with optimal drug-to-antibody ratio, efficient tumor killing potency, and favorable safety profiles

**DOI:** 10.1371/journal.pone.0326691

**Published:** 2025-06-26

**Authors:** Ting Zhang, Jianyan Xu, Junzhao Yin, Yun Gao, Hanwen Zheng, Beibei Fu, Jiakang Sun, Zhibin Xu, Shiwei Tu, Yuchang Mao, Weiyun Wen, Bolei Qu, Lingfeng You, Zhendong Xue, Xing Sun, Dan Cao, Jun Feng, Min Hu, Feng He

**Affiliations:** 1 Shanghai Hengrui Pharmaceutical Co., Ltd., Shanghai, China; 2 Shanghai Shengdi Pharmaceutical Co., Ltd., Shanghai, China; China Medical University (Taiwan), TAIWAN

## Abstract

HER2-targeting antibody–drug conjugates (ADCs), especially trastuzumab deruxtecan (T-DXd), have revolutionized the treatment landscape of HER2-expressing or mutant cancers. However, undesired adverse events are still inevitable and it is necessary to discover a HER2-directed ADC with better safety profiles. SHR-A1811 is composed of trastuzumab, a cleavable linker and a novel topoisomerase I inhibitor, SHR169265. The results indicated that SHR169265 shows better permeability, strong cytotoxicity and faster systemic clearance than DXd analog (SHR197971). The drug-to-antibody ratio (DAR) of SHR-A1811 was optimized as 6 via balancing efficacy and toxicity. SHR-A1811 showed HER2-dependent growth inhibition against various cell lines and desirable bystander killing capability. SHR-A1811 led to tumor growth inhibition or even regression in a dose-dependent manner, at least comparable as HRA18-C015 (a synthesized T-DXd) and anti-HER2-SHR169265 (DAR 8) in multiple mouse xenograft models with a range of HER2 expression levels. SHR-A1811 exhibited a good pharmacokinetics profile, outstanding stability in plasma across different species and a favorable preclinical safety profile. The highest non-severely toxic dose (HNSTD) in cynomolgus monkeys was 40 mg/kg with thymus as the main target organ. The above results suggested that SHR-A1811 is a potential best-in-class anti-HER2 ADC with a highly permeable payload, optimized DAR, great potency and better safety profiles. Currently SHR-A1811 has entered phase II and phase III clinical studies for breast cancer, gastric cancer, colorectal cancer, and NSCLC.

## Introduction

Human epidermal growth factor receptor 2 (ERBB2, HER2) is a well-validated proto-oncogene, overexpressed, amplified or mutated in multiple advanced solid tumors [1–3]. Trastuzumab deruxtecan (T-DXd, DS-8201a, ENHERTU), a representative of the third-generation ADC, has been approved for the treatment of HER2-expressing breast cancer, gastric cancer and HER2 mutant non-small cell lung cancer (NSCLC) [4–7]. It is structurally composed of the HER2 antibody trastuzumab, a cleavable peptide-based linker and a topoisomerase I inhibitor payload MAAA-1181a (DXd, exatecan derivative) [8,9]. The high drug load (DAR 8) and bystander killing effect made T-DXd the first HER2-targeted therapy showing clinical benefit in HER2 low-expressing (IHC 1+ or IHC 2 + /FISH-) breast cancer [5,8–10]. However, although a stable linker and high systemic clearance of payload were designed to minimize toxicities, the high rate of associated adverse effects, such as hematologic and gastrointestinal disorders, left ventricular dysfunction and interstitial lung disease (ILD)/pneumonitis, pointed to a room for further improvement [11,12].

T-DXd has been issued a black box warning for the risk of ILD/ pneumonitis [13]. Permanent treatment cessation is recommended with grade 2 or higher ILD/pneumonitis [14]. The pooled clinical data analysis (DS8201-A-J101, DESTINY-Breast01, DESTINY-Breast03, DESTINY-Breast04, and DESTINY-Lung02) suggested that the incidence of ILD/pneumonitis was 12% in patients treated with 5.4 mg/kg of T-DXd [5,14–17]. In the global phase III study in HER2-positive metastatic breast cancer patients (DESTINY-Breast03), where patients received 5.4 mg/kg of T-DXd, treatment discontinuation occurred in 14% of patients and ILD/pneumonitis accounted for 8%. Moreover, the dose interruption and reduction rates in T-DXd arm were 44% and 21%, respectively, mainly due to neutropenia, leukopenia, and nausea, *etc*. The incidence of drug-related adverse events of grade 3 or 4 reached 45.1% [18]. In DESTINY-Gastric01 trial, 15% of discontinuation rate was observed in patients receiving 6.4 mg/kg of T-DXd, and ILD/pneumonitis accounted for 6%. Dose interruption occurred in up to 62% of patients [6]. Therefore, it is necessary to discover a novel anti-HER2 ADC with better safety margin, without jeopardizing the efficacy.

In this study, we described SHR-A1811, a HER2-ADC consisting of trastuzumab, a cleavable linker and a unique topoisomerase I inhibitor payload SHR169265. SHR169265 was designed to have potent cytotoxicity and desirable bystander killing effect to ensure a comparable antitumor efficacy of SHR-A1811 as T-DXd, even though with lower DAR value of 6. Most importantly, the chemical modification of SHR169265 improved the plasma stability of SHR-A1811. Optimized DAR and high stability together led to potential low free toxin exposure *in vivo* and optimal safety profiles in GLP toxicity study in cynomolgus monkeys. Collectively, these favorable preclinical profiles supported SHR-A1811 a potential best-in-class HER2-targeting ADC, and multiple clinical trials are ongoing in patients with HER2-expressing and/or mutant cancers.

## Materials and methods

### Antibody–drug conjugates and chemicals

SHR167971 was synthesized using published DXd structure [8]. Trastuzumab was produced with the published amino acid sequence [19]. HRA18-C015 and HRA18-K001 were synthesized according to the published T-DXd and T-DM1 structures, respectively [8,20]. Anti-HER2-SHR169265 (DAR 4), SHR-A1811, and anti-HER2-SHR169265 (DAR 8) were composed of trastuzumab, a maleimide glycine-glycine-phenylalanine-glycine (GGFG) peptide linker and SHR169265 with the DAR values at 4, 6 and 8, respectively. Human IgG1-ADC was composed of isotype control IgG1, GGFG linker and SHR169265 with DAR at 8. The four inter-strand disulfide bonds of antibodies were reduced to eight cysteine residues by tris(2-carboxyethyl) phosphine. The linker and payload were connected as described previously [21]. Linker-payload was conjugated to the cysteine residues using a conventional strategy [22].

### Lipophilicity assay

Distribution coefficient (LogD, pH 7.4) was obtained by a high-throughput alternative method [23]. Each compound was dissolved in DMSO at 10 mM. After centrifugation, the solution was measured by reversed-phase HPLC on a C18 column (50 × 4.6 mm, 5 µM Gemini C18, Phenomenex) at pH 7.4, using fast gradient acetonitrile-aqueous buffer mobile phases. The lipophilicity was estimated from a calibration curve using six reference compounds by plotting their respectively measured retention time versus LogD in literature. The chromatographic LogD values were derived directly from the gradient retention time by using this calibration curve.

### Parallel artificial membrane permeability (PAMPA) assay

PAMPA assay was outsourced to Wuxi Apptech Co. Ltd. Briefly, donor plate, acceptor plate and receiver plate formed a sandwich and 200 µL of 5.0 µM donor solutions were added. The concentrations of compounds in both plates were measured by a LC-MS/MS system after 30 minutes. The permeability coefficient (Peff, nm/s) values at pH 5 and 7.4 were calculated.

### Topoisomerase I inhibition assay

Topoisomerase I inhibition assay was outsourced to Wuxi Apptech Co. Ltd. Topoisomerase I activity was measured using human topoisomerase I microplate assay kit (Inspiralis), according to the manufacturer’s instructions.

### Pharmacokinetics of payload in mice

All animal experiments at Shanghai Hengrui were approved by Shanghai Hengrui’s Institutional Animal Care and Use Committee. Studies were performed in accordance with ARRIVE guidelines. C57BL/6 mice (15–18 g body weight, n = 18) were purchased from Beijing Vital River Laboratory Animal Technology Co., Ltd and were held as described in details of mouse xenograft section. Compounds were administered intravenously at 0.5 mg/kg. Blood samples were collected at various time points after dosing. Mice were monitored daily for health condition and humane endpoint criteria. There were no unexpected animal deaths or humane endpoint criteria reached during the study. All of the animals were euthanized by using inhaled carbon dioxide at the end of study. The compound concentrations were determined by SCIEX Triple Quad 7500 LC-MS/MS System (SCIEX).

### Cell lines

The human breast cancer cell lines SK-BR-3, HCC1954, and MDA-MB-468, the human gastric carcinoma cell lines NCI-N87, AGS, and ANU-16, and the human pancreatic cancer cell line Capan-1 were purchased from American Type Culture Collection. The human breast cancer cell line JIMT-1 was purchased from Cobioer Biosciences Co. LTD. The human gastric carcinoma cell line MKN-45 was purchased from Japanese Collection of Research Bioresources Cell Bank. MDA-MB-468 cell line was cultured in Leibovitz’s L-15 medium (Gibco) supplemented with 10% FBS (Gibco) at 37°C without CO_2_. All other cell lines were cultured with appropriate medium (DMEM (Gibco) for JIMT-1; RPMI1640 (Gibco) for HCC1954, NCI-N87, AGS, SNU-16, and MKN-45; McCoy’s 5A (Gibco) for SK-BR-3; and IMDM (Gibco) for Capan-1) supplemented with 10% FBS at 37°C under 5% CO_2_ atmosphere.

### Flow cytometry analysis of HER2 expression

2 × 10^5^ cells were incubated with BB700 mouse anti-human HER2 antibody (BD Biosciences) at 1 μg/mL for 30 minutes at 4°C. The fluorescent signal was detected using BD FACSVerse. The data was analyzed by FlowJo_v10.8.1 (BD Biosciences).

### Cell viability assay

Cells were seeded into 96-well plates at 1500 cells/well for SK-BR-3, JIMT-1, and NCI-N87, 800 cells/well for HCC1954, 600 cells/well for AGS and MKN-45, 2500 cells/well for MDA-MB-468 and Capan-1, and 1000 cells/well for SNU-16. After overnight incubation, a serially diluted solution of compounds or ADCs was added. Cell viability was evaluated after 6 days using CellTiter-Glo reagent (Promega) according to the manufacturer’s instructions. Luminescence was measured by PHERAstar (BMG LABTECH).

### Western blot analysis

SK-BR-3 cells were incubated with 2 nM of ADCs for 48 hours and then lysed in lysis buffer (Cell Signaling Technology) containing PMSF and PhosSTOP (Roche). Twenty to Sixty microgram of protein lysates were separated by SDS-PAGE gel and electro-transferred to polyvinylidene difluoride membranes. Blots were probed by anti-cleaved PARP (Asp214) antibody (1:1000 dilution, Cat#: 9541, Cell Signaling Technology), anti-phospho-Histone H2A.X (Ser139) antibody (1:1000 dilution, Cat#: 9718, Cell Signaling Technology), anti-GAPDH (D16H11) XP Rabbit antibody (1:1000 dilution, Cat#: 5174, Cell Signaling Technology), and anti-HSP70 antibody (1:1000 dilution, Cat#: 4872, Cell Signaling Technology). The membranes were subsequently incubated with secondary antibody (anti-rabbit IgG, 1:1000 dilution, Cat#:7074, Cell Signaling Technology) and Supersignal kit (Thermo Scientific). The chemiluminescence signal was detected and quantified using a ChemiDoc MP imaging System (Bio-Rad).

### Caspase-Glo 3/7 assay

SK-BR-3 cells were treated with a serially diluted solution of ADCs for 3 days. The Caspase-Glo™ 3/7 reagent (Promega) was added according to manufacturer’s guidelines. Luminescence was measured by PHERAstar (BMG LABTECH).

### Bystander killing effect

HER2-positive SK-BR-3 and HER2-negative MDA-MB-468 cells were seeded together into 6-well plates at 1.5 × 10^5^ cells/well and 1 × 10^5^ cells/well, respectively. Cells were treated with ADCs for 3 or 5 days. In order to determine the ratio of SK-BR-3 and MDA-MB-468 in each well, cells were harvested at endpoint to count total number and stained with BB700 mouse anti-human HER2 antibody (BD Biosciences). The fluorescent signal of stained cells was analysed using BD FACSVerse. Based on the ratio of positive *vs.* negative cells, the residual numbers of SK-BR-3 and MDA-MB-468 cells in each well were calculated.

### Mouse xenograft experiments

All the animal procedures have been approved by the Institutional Animal Care and Use Committee (IACUC) of Shanghai Hengrui Pharmaceutical Company. Mice were acclimated to the housing environment for at least one week before any procedure. All the studies were carried out in temperature- and humidity-controlled barrier facility. The temperature was maintained at 20–26 degree Celsius, and the relative humidity 40–70%. All the animal holding rooms were on a “12 hour on” (7 am to 7 pm) and “12 hour off” (7 pm to 7 am) light cycle. Mice were group housed with commercially available corncob bedding and environmental enrichment. Commercially irradiated rodent diet and autoclaved tap water were ad libitum. All of the research staff have been trained for animal handling, restrain, dosing, and proper euthanasia technique.

Female NUNU nude mice, Beige SCID mice and Balb/c nude mice (15–18 g body weight, n = 385) were purchased from Beijing Vital River Laboratory Animal Technology Co., Ltd. In brief, NCI-N87 (5 × 10^6^ cells/inoculation), JIMT-1 (4.5 × 10^6^ cells/inoculation) and Capan-1 (3 × 10^6^ cells/inoculation) were implanted subcutaneously into the hind flank region and allowed to grow to the appropriate size (150–250 mm^3^ in average). Six to eight mice per treatment group were randomized according to the size of tumors. A head-to-head PK analysis between intraperitoneal and intravenous administration of SHR-A1811 and HRA18-C015 was performed. These two dosing regimens revealed comparable AUC and half-life (S1 Table). The ADCs were administered intraperitoneally at various dosages single time or every week for two weeks as described in the figure legend. Tumor volume and body weight were measured twice a week during the course of the study. Tumor growth inhibition (TGI, %) was calculated as TGI% = [1-(T-T_0_)/(C-C_0_)] x 100%, where C_0_ and T_0_ stand for the average tumor volume of vehicle and treatment groups at study start, and C and T for the average tumor volume of vehicle and treatment groups at endpoint. Tumor volume of individual tumors at each time point was measured as V = 1/2 x Length x Width^2^. Mice were examined daily for health condition and humane endpoint criteria. Animals were sacrificed in less than a day once criteria was reached. There were no unexpected animal deaths during the *in vivo* studies with 40–50-day duration. All of the mice were euthanized using inhaled carbon dioxide at the end of study or when the following humane endpoints were reached: tumor volume exceeding 2000 mm^3^, body weight decreases by more than 20% of the initial weight, tumor ulceration, any sign of severe pain distress or infection.

### Immunohistochemistry

NCI-N87, JIMT-1 and Capan-1 xenografted tumors were harvested and fixed in 10% neutral buffered formalin and embedded in paraffin. For antigen retrieval, the sections were incubated at 100°C for 15 minutes in EDTA buffer (pH 9.0). After blocking of nonspecific reactions by 1.5% BSA, the sections were incubated with anti-Her2/neu antibody (clone SP3; 1:100; Invitrogen) for 60 minutes at room temperature. The sections were then washed and incubated with polymer-based signal amplification system (ImmPRESS® HRP Horse Anti-Rabbit IgG Polymer Detection Kit, Vector Laboratories) for 30 minutes. The 0.01% diaminobenzidine (DAB, MaiXin Biotech) was used to develop a brown chromogen, and the mounted slides were scanned.

### PK/PD study

In the NCI-N87 xenograft model, blood samples and tumor tissues were collected at 8, 24, 72 and 168 hours after single dose of ADCs. The cleaved PARP levels in tumors were detected using western blotting. The concentrations of payload in tumor and blood samples were detected by SCIEX Triple Quad 7500 LC-MS/MS System (SCIEX).

### Pharmacokinetics in rats

All the animal procedures have been approved by the Institutional Animal Care and Use Committee (IACUC) of Shanghai Hengrui Pharmaceutical Company. Rats were acclimated to the housing environment for at least 5-days before any procedure. All the studies were carried out in temperature- and humidity-controlled barrier facility. The temperature was maintained at 20–26 degree Celsius, and the relative humidity 40–70%. All the animal holding rooms were on a “12 hour on” (7 am to 7 pm) and “12 hour off” (7 pm to 7 am) light cycle. Rats were group housed with commercially available corncob bedding and environmental enrichment. Commercially irradiated rodent diet and autoclaved tap water were ad libitum. Animal handling, proper injection for treatment/anesthesia, blood collection, and painless death were taught to the staff.

SD rats (160–180 g body weight, n = 4) were purchased from Beijing Vital River Laboratory Animal Technology Co., Ltd. After an adaptation period, 3 mg/kg of SHR-A1811 was intravenously administered. Blood sample was collected at various time points after dosing. The concentration of total antibody and intact ADC were determined by HTRF method. Clinical and humane endpoint criteria were observed twice a day during study. Rats were sacrificed in less than a day once the endpoint was reached. There were no unexpected animal deaths during the *in viv*o studies with 4-week duration. All of the rats were euthanized using inhaled carbon dioxide at the end of study or when the following humane endpoints were reached: bodyweight decreases by more than 20% of the initial weight, persistent anorexia, or any sign of severe pain distress.

### *In vitro* stability of SHR-A1811 in plasma

20 μg/mL of SHR-A1811 was incubated in mouse, rat, monkey, and human plasma at 37°C for up to 21 days. The concentrations of released payload were quantified by SCIEX Triple Quad 7500 LC-MS/MS System (SCIEX). The released payload proportional to the theoretical concentration of all payload in ADC was calculated as: release rate (%) = C_payload_/(C_ADC_ x DAR) x 100%, where C stands for concentration (nM).

### GLP toxicology and toxicokinetics studies in cynomolgus monkeys

GLP toxicology and toxicokinetics studies were outsourced to Shanghai InnoStar Bio-tech Co., Ltd. Studies were approved by InnoStar’s Institutional Animal Care and Use Committee and carried out in compliance with ARRIVE guidelines. Cynomolgus monkeys were housed in stainless steel cages at the study institution. The temperature and relative humidity of the room were recorded twice a day with ranged from 18 to 26°C and 40–70%, respectively. Ventilation was constantly operated with ≥ 8 times of air changes per hour. All cynomolgus monkey holding rooms were on a 12-h cycle. Monkey maintenance feed was purchased from qualified supplier with microorganisms, heavy metals and pesticide residues tested. During the study, all animals had ad libitum access to feed and clean deionized water except during specific fasting. The test results for both feed and drinking water showed no potential contamination that could affect the study results. All animals were acclimated to temperature and relative humidity-controlled environment for few days before randomization. Detailed clinical observation was performed on all cynomolgus monkeys once a day during the acclimation period. All of the animal care staff were trained to maximal reduce the pain of animals. Special training such as animal handling, proper injection for treatment/anesthesia, and painless death were taught to the staff.

Briefly, SHR-A1811 was intravenously administered every 3 weeks for a total of 5 doses in cynomolgus monkeys (N = 10 for each group, 5 animals per sex) followed by 4-week recovery. Clinical signs, body weight, food consumption, and clinical pathology were monitored twice a day. The blood samples were collected after the first and fourth doses of 40 mg/kg to measure the concentrations of SHR-A1811, total antibody and free payload. Humane endpoint criteria were observed twice a day during study to examine health and behavior. Animals were sacrificed in less than a day once reached endpoint criteria. There were no unexpected animal deaths during the studies with 16-weeks duration. All of the animals were euthanized by intravenous injection of combination of zoletil and xylazine at the end of study for targeted organ and tissue examinations to evaluate toxicity or when the following humane endpoints were reached: severe injury, severe respiratory depression, severe weight loss, persistent anorexia, significant pain or stress, progressive pain leading to weakness, and persistent or continuous twitching. Final disposal of each animal was recorded on the study records.

### Statistics analysis

Data are presented as means ± SEM from at least three independent experiments. GraphPad Prism software version 9 (GraphPad Software) was used for curve fitting. Differences between two groups were analyzed by a standard Student t-test. For multigroup comparisons, two-way ANOVA were performed. P < 0.05 was considered statistically significant.

### Ethics statement

Ethics approval and consent to participate animal experiments at Shanghai Hengrui were approved with records by the Institutional Animal Care and Use Committee of Shanghai Hengrui (Reference number: 20210317-mouse-1, 20210317-mouse-4, 20210317-mouse-6). Animal studies at Shanghai InnoStar was approved with records by InnoStar’s Institutional Animal Care and Use Committee (Reference number: IACUC-2021-M-029). All animal studies were carried out in accordance with ARRIVE guidelines.

## Results

### Physicochemical characteristics, topoisomerase I inhibition and pharmacokinetic profile of SHR169265

**Fig 1:**
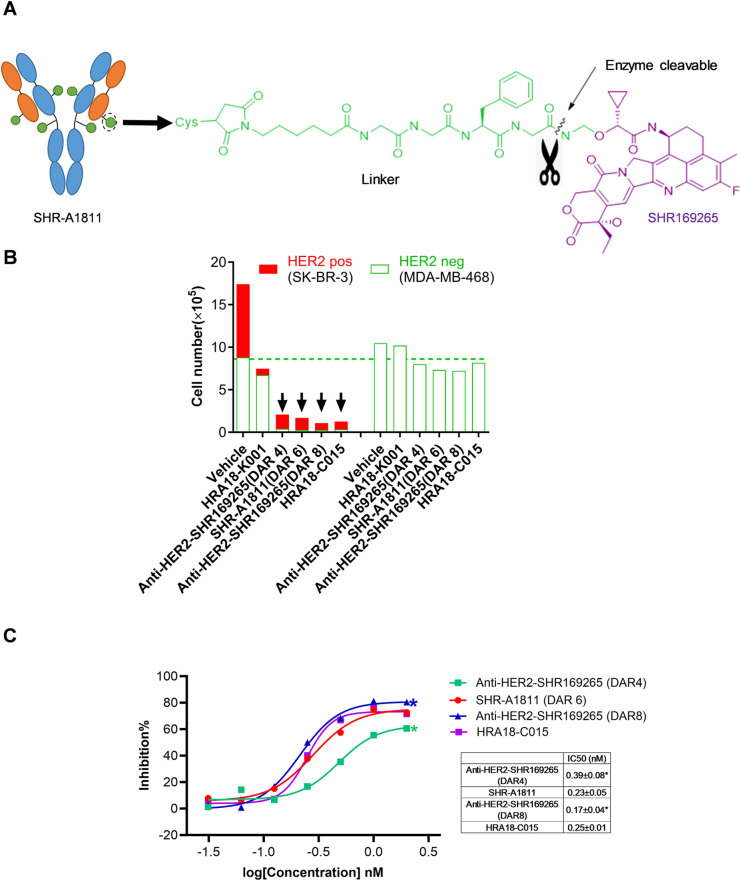
Structure and bystander killing effect of anti-HER2-SHR169265 ADCs. **(A)** Structure illustration of SHR-A1811. SHR-A1811 is composed of trastuzumab, a stable and cleavable linker, and a topoisomerase I inhibitor payload SHR169265, with DAR at 6. **(B)** Bystander killing effect of anti-HER2-SHR169265 ADCs. SK-BR-3 (HER2-positive) and MDA-MB-468 (HER2-negative) cells were co-cultured and treated. For comparison, MDA-MB-468 cells were cultured alone and treated in the same way. Cell numbers of SK-BR-3 and MDA-MB-468 cells after treatment with 10 nM of ADCs for 5 days were analyzed. **(C)** The inhibitory effect of ADCs on MDA-MB-468 in the co-culture system for bystander killing. Cells were treated with various doses of ADCs for 3 days. Data represented three independent experiments. *P < 0.05 versus HRA18-C015.

SHR169265 (Fig 1A) is an exatecan derivative with a chiral cyclopropyl at the carbonyl alpha position [21,24], selected based on parameters including potency, solubility, permeability, and pharmacokinetics. The lipophilicity and permeability of SHR169265 were evaluated by LogD (pH 7.4), ALogP, and Peff values. The LogD (pH 7.4) and ALogP values showed that SHR169265 had moderate lipophilicity. The Peff values at pH 5 and pH 7.4 of SHR169265 were 137.7 and 71.6 nm/s, respectively, about 5 times higher than those of SHR167971 (Table 1), indicating that SHR169265 had high membrane permeability. The inhibition of SHR169265 on DNA topoisomerase I enzymatic activity was comparable with that of SHR167971 (S1 Fig). In addition, SHR169265 showed faster systemic clearance in mice than SHR167971 (CL: 72.6 *vs*. 54 mL/min/kg; 0.5 mg/kg, i.v.).

**Table 1:** Physicochemical parameters, topoisomerase I inhibitory activity and pharmacokinetic parameters of SHR169265.

		SHR169265	SHR167971^a^
Molecule weight		533.55	493.49
LogD (pH 7.4)		1.89	1.43
AlogP (calc.)		3.67	2.72
PAMPA Peff (nm/s)	pH 5	137.7	25.9
	pH 7.4	71.6	17.5
Top I inhibition (IC_50_, μM)		1.34	0.97
PK parameters in mice (0.5 mg/kg, i.v. injection)	Cmax (ng/mL)	250	343
	AUC_0-t_ (ng/mL*h)	113	154
	t1/2 (h)	0.37	0.27
	CL (mL/min/kg)	72.6	54
	Vss (mL/kg)	1815	1276

^a^SHR167971: payload synthesized with published DXd structure.

### Cytotoxicity of SHR169265

The cytotoxicity of SHR169265 was evaluated in nine cancer cell lines. Overall, SHR169265 exhibited comparable or slightly better cell growth inhibition activity with SHR167971 across cancer cell lines (Table 2). SHR169265 induced strong cytotoxicity effect with 0.3-2.32nM IC_50_ values independent of HER2 expression levels.

**Table 2:** Cytotoxicity of SHR169265 and anti-HER2-SHR169265 ADCs.

IC_50_ (nM)	HER2 level	SHR169265	SHR167971	Anti-HER2-SHR169265 (DAR 4)	SHR-A1811 (DAR 6)	Anti-HER2-SHR169265 (DAR 8)	HRA18-C015^a^ (DAR 8)	Control IgG1-ADC (DAR 8)	Trastuzumab
NCI-N87	High	1.00 ± 0.07	2.91 ± 0.53	0.85 ± 0.19	0.57 ± 0.15	0.37 ± 0.11	0.30 ± 0.04	>100	>100
SK-BR-3	High	0.58 ± 0.10	1.38 ± 0.23	0.66 ± 0.13	0.42 ± 0.10	0.28 ± 0.09	0.29 ± 0.15	>10	>3
HCC1954	High	0.55 ± 0.19	1.07 ± 0.35	1.28 ± 0.15	0.77 ± 0.10	0.44 ± 0.17	0.50 ± 0.09	>100	>500
JIMT-1	Medium	2.32 ± 0.45	7.85 ± 2.96	495.4 ± 65.5	302.9 ± 87	260.2 ± 12	203.8 ± 8.6	1230 ± 181.7	>5000
Capan-1	Low	0.38 ± 0.07	1.29 ± 0.41	38.4 ± 7.7	11.3 ± 2.7	5.4 ± 1.7	11.2 ± 0.9	57.2 ± 10.3	>500
AGS	Low	0.36 ± 0.11	0.60 ± 0.27	107.8 ± 24.5	59.1 ± 17.2	47.3 ± 8.1	30.3 ± 8.4	68.4 ± 25.1	>500
MKN45	Low	0.30 ± 0.07	1.18 ± 0.22	82.5 ± 14.1	42.5 ± 7.8	38.5 ± 6.6	35.9 ± 6.8	74.5 ± 24.2	>500
SNU-16	Low	0.57 ± 0.19	1.58 ± 0.25	161.8 ± 5.8	132.6 ± 22.3	88.8 ± 16.2	74.8 ± 18.6	168.3 ± 27.3	>500
MDA-MB −468	Negative	0.56 ± 0.09	1.13 ± 0.14	146.6 ± 30.5	97.3 ± 23.1	86.8 ± 16.7	59.1 ± 12.5	206.9 ± 44.6	>500

^a^HRA18-C015: a synthesized T-DXd

### Cell killing activity of anti-HER2-SHR169265 ADCs

Three anti-HER2-SHR169265 ADCs composed of trastuzumab, a cleavable linker and payload SHR169265 with DAR values at 4, 6, and 8 were synthesized. The linker was a maleimide glycine-glycine-phenylalanine-glycine (GGFG) peptide linker and can be cleaved by the enzymes in lysosome. After endocytosis, the ADCs are cleaved at the indicated position, and the first intermediate undergoes self-degradation to release the payload SHR169265 (Fig 1A).

Nine breast cancer and gastric cancer cell lines were divided into 4 groups, HER2-high (NCI-N87, SK-BR-3, and HCC1954), HER2-medium (JIMT-1), HER2-low (capan-1, AGS, MKN45, and SNU-16) and HER2-negative group (MDA-MB-468), according to the flow cytometry analysis (S2 Fig). Compared to Trastuzumab which required high antibody concentration to achieve cytotoxicity in tumor cells, anti-HER2-SHR169265 ADCs induced much stronger cytotoxicity in HER2-expressing cells without obvious effect on HER2-negative cells (MDA-MB-468). This inhibitory capacity was in general correlated with DAR value (Table 2). It was also correlated with HER2 expression levels, except that JIMT-1 cell line was an outlier, probably due to its relative insensitivity to SHR169265 (Table 2). SHR-A1811 (DAR 6) showed slightly weaker potency (1.2–1.5 fold less) than anti-HER2-SHR169265 (DAR 8) and HRA18-C015 (DAR 8), attributable to lower DAR.

To confirm the mechanism of cytotoxicity was due to inhibition of DNA topoisomerase I, the DNA damage markers (histone H2A.X phosphorylation) and apoptosis markers (cleaved PARP and caspase 3/7 levels) were analyzed on SK-BR-3 cells. All three anti-HER2-SHR169265 ADCs and HRA18-C015 significantly increased these markers (S3 Fig). The degree of caspase 3/7 induction was correlated with DAR values.

### Bystander killing effect of anti-HER2-SHR169265 ADCs

To evaluate the bystander killing effect of ADCs, HER2-positive cells (SK-BR-3) and HER2-negative cells (MDA-MB-468) were co-cultured. As expected, HRA18-K001 (a synthesized T-DM1) mainly inhibited HER2-positive cell growth, suggesting a poor bystander killing capacity as reported [10]. In contrast, three anti-HER2-SHR169265 ADCs and HRA18-C015 killed both HER2-positive cells and their adjacent negative cells at 10 nM (Fig 1B), without much effect on HER2-negative cells when cultured alone. The bystander killing IC_50_ on MDA-MB-468 cells were further analyzed in the co-culture system (Fig 1C). Among all tested ADCs, anti-HER2-SHR169265 (DAR 8) exhibited the strongest bystander killing effect (IC_50_, 0.17 ± 0.04 nM). SHR-A1811(DAR 6) and HRA18-C015 (DAR 8) showed a comparable bystander killing effect (IC_50_, 0.23 ± 0.05 *vs.* 0.25 ± 0.01 nM), embodying the advantage of high membrane permeability of payload.

### Antitumor efficacy of anti-HER2-SHR169265 ADCs and DAR optimization

**Fig 2:**
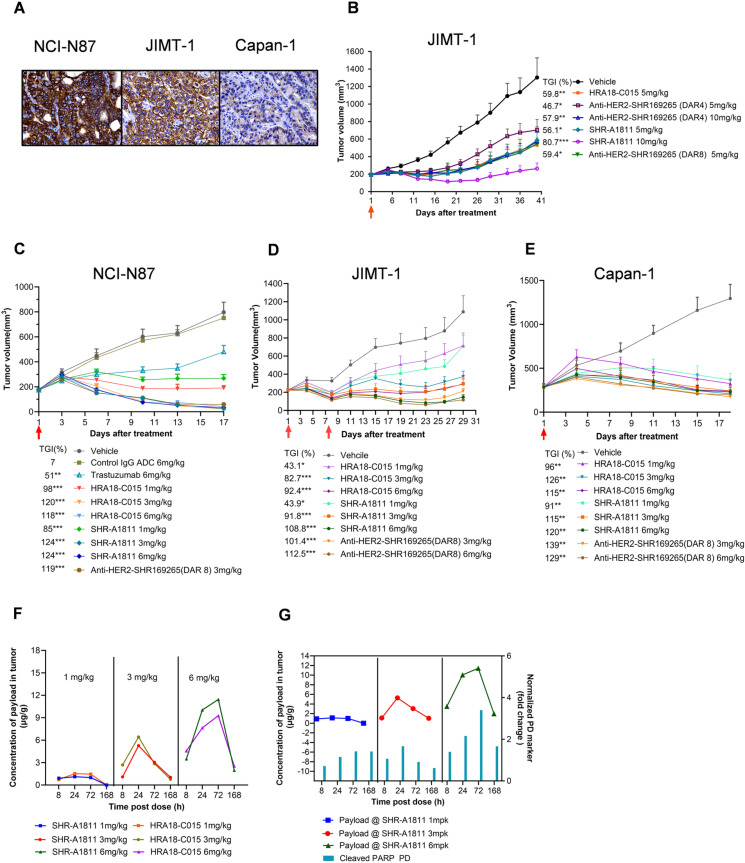
Antitumor effect of anti-HER2-SHR169265 ADCs. **(A)** HER2 IHC staining on NCI-N87, JIMT-1 and Capan-1 xenografted tumors. **(B)** Comparison of antitumor effect of anti-HER2-SHR169265 ADCs with different DAR values in JIMT-1 xenograft model. **(C-E)** Antitumor effect of SHR-A1811 in NCI-N87 (C), JIMT-1 (D) and Capan-1 (E) xenograft models. All of the tumor-bearing mice were intraperitoneally administered with ADCs single dose except JIMT-1 xenograft model in panel D, with every week administration for two weeks. Arrows indicate days of administration. The mean tumor volume and SEM (n = 6-8) were represented on the graph, and TGI% calculated for each treatment group. *P < 0.05, **P < 0.01, ***P < 0.001 versus vehicle. **(F)** The concentration of SHR169265 and SHR167971 in NCI-N87 tumors after single dose of SHAR-A1811 and HRA18-C015. **(G)** PK/PD relationship of SHR-A1811 in NCI-N87 model. The tumoral SHR169265 concentration and cleaved PARP level were measured at 8, 24, 72 and 168 hours after single dose of ADC. Data shown as means from two tumor samples.

In order to determine the optimal DAR value, the antitumor efficacy of three anti-HER2-SHR169265 ADCs was evaluated in HER2-moderate JIMT-1 xenograft models (Fig 2A) after single dose. At the dosage of 5 mg/kg, SHR-A1811 (DAR 6) led to 57.9% TGI, comparable with HRA18-C015 (59.8% TGI) and anti-HER2-SHR169265 (DAR 8) (59.4% TGI), and stronger than anti-HER2-SHR169265 (DAR 4) (46.7% TGI). The difference between SHR-A1811 and anti-HER2-SHR169265 (DAR 4) was further amplified at 10 mg/kg (TGI 80.7% *vs.* 57.9%) (Fig 2B). Considering the overall *in vitro* and *in vivo* profiles, SHR-A1811 and anti-HER2-SHR169265 (DAR 8) were chosen for further evaluation to fine tune the DAR.

Three xenograft models (NCI-N87, JIMT-1 and Capan-1) with a range of HER2 expression levels were established (Fig 2A). SHR-A1811 inhibited tumor growth in a dose-dependent manner in all three models (Figs 2C-E), without effect on mouse body weight (S4 Fig). In NCI-N87 (HER2-high) and Capan-1 (HER2-low) models, the TGIs of SHR-A1811, HRA18-C015 and anti-HER2-SHR169265 (DAR 8) were comparable at the same dosage. Tumor regression was observed at 3 mg/kg of SHR-A1811 and sustained till day 17 (124% TGI in NCI-N87 model; 115% TGI in Capan-1 model). Trastuzumab at 6 mg/kg caused 51% of TGI in NCI-N87 model. Control IgG1-ADC showed no effect on tumor growth. In JIMT-1 (HER2-moderate) model, the TGIs of SHR-A1811 and anti-HER2-SHR169265 (DAR 8) were greater than that of HRA18-C015 at 3 and 6 mg/kg after 2 doses. In this model, multiple dosing could better embody the advantages of SHR-A1811 and anti-HER2-SHR169265 (DAR 8), comparing to the results of single dose (Fig 2B). All these data demonstrated that SHR-A1811, though with 25% less payload, conferred at least comparable antitumor activity as HRA18-C015. The good membrane permeability of SHR169265 could bring in a strong bystander effect in solid tumors, contributing to the potent antitumor efficacy. Since the lower DAR used in SHR-A1811 can potentially elicit lower side effects without compromising the efficient tumor killing potency, we finally chose SHR-A1811 (DAR 6) as our lead molecule.

PD biomarkers and released payload in plasma and NCI-N87 tumors were analyzed after single dose of SHR-A1811 or HRA18-C015. As shown in Fig 2F, free payload was detected in tumors and the concentrations were proportional to the dosages of ADCs. At 3 and 6 mg/kg, the tumoral SHR169265 levels reached Cmax at 24 and 72 hours after treatment, respectively. The tumoral AUC of SHR169265 and SHR-167971 were comparable at the same dosage. Consistent with the stability of ADCs and fast clearance of free payload in circulation, the levels of payloads in plasma were below the limitation of quantitation (0.1 ng/mL). In addition, SHR-A1811 treatment induced PARP cleavage in tumor cells. The degree of PD marker changes was correlated with tumoral SHR169265 levels (Fig 2G).

### Pharmacokinetics profile and plasma stability of SHR-A1811

**Fig 3:**
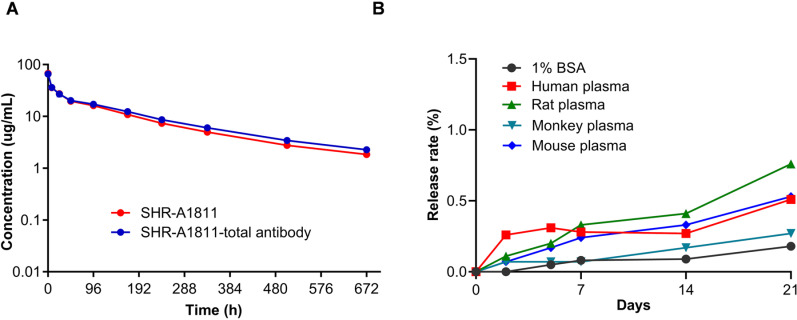
Pharmacokinetics and plasma stability of SHR-A1811. **(A)** Pharmacokinetics of SHR-A1811 in rats. SHR-A1811 were intravenously administered at the dose of 3 mg/kg. Concentrations of SHR-A1811 and total antibody were measured. **(B)** *In vitro* stability of SHR-A1811 in plasma of different species. SHR-A1811 was incubated in mouse, rat, monkey, and human plasma at 37°C for up to 21 days. The concentrations of released payload were detected, and release rates (%) was calculated.

After single intravenous injection of SHR-A1811 at 3 mg/kg in rats, the exposure levels of ADC and total antibody were measured. The PK profiles of SHR-A1811 and total antibody were similar (t1/2: 8.3 and 8.7 days; AUC: 5598 and 6156 μg.h/mL; Clearance: 11.8 and 10.6 mL/day/kg, respectively, Fig 3A), suggesting the stability of the linker-payload system *in vivo*.

The stability of SHR-A1811 was also evaluated in plasma incubation system. The release rate of SHR169265 from SHR-A1811 ranged from 0.27% to 0.76% after 21-day incubation in mouse, rat, monkey and human plasma (Fig 3B), indicating an excellent *in vitro* plasma stability. Taken together, the *in vitro* and *in vivo* results confirmed the high stability of SHR-A1811 and the potential low free toxin exposure in circulation.

### Safety profiles and toxicokinetics of SHR-A1811

**Fig 4:**
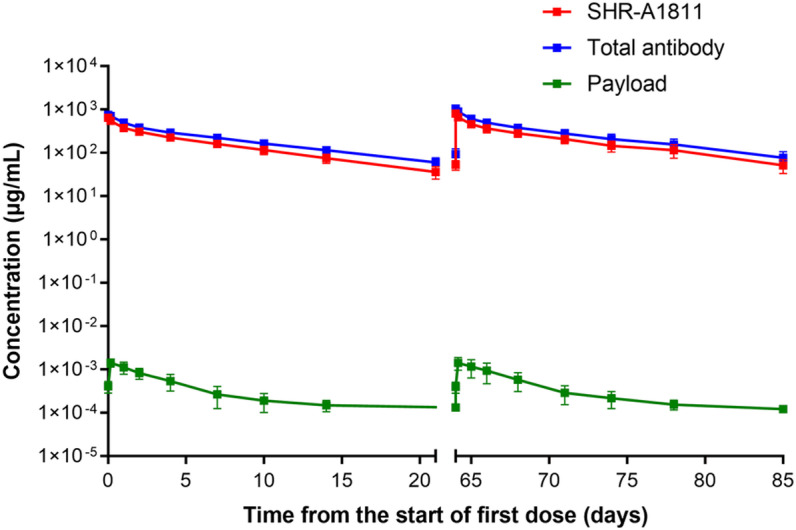
Toxicokinetics of SHR-A1811 in cynomolgus monkeys. SHR-A1811 was intravenously administered at the dose of 40 mg/kg every 3 weeks for 5 doses (n = 10). SHR-A1811, total antibody and payload concentrations were measured after the first and fourth doses. Data shown as means from ten samples.

The safety of multiple administration (every 3 weeks for 5 doses) of 3, 10, and 40 mg/kg SHR-A1811 was evaluated in cynomolgus monkeys. SHR-A1811 reduced reticulocyte number and percentage by 80%, thymus weight and organ coefficient by 71% at 40 mg/kg. All adverse effects recovered after 4 weeks of drug withdrawal. No clinical abnormalities were observed at 3 or 10 mg/kg. Therefore, the HNSTD of SHR-A1811 in cynomolgus monkeys was determined as 40 mg/kg with thymus as the main target organ (Table 3). The serum concentrations of SHR-A1811, total antibody and payload were measured after the first and fourth doses of 40 mg/kg (Fig 4 and Table 4). The toxicokinetics parameters of SHR-A1811 after single and multiple does were comparable, suggesting low immunogenicity. Consistent with the pharmacokinetics of SHR-A1811 in rats, the TK profile of SHR-A1811 was similar with that of total antibody after multiple doses (t1/2: 7.1 and 7.5 days; AUC_t_: 3988 and 5452 day*μg/mL, respectively) in cynomolgus monkeys. Most importantly, only very small amount of free toxin was detected in plasma. Compare to the high Cmax of SHR-A1811 (801.1 µg/mL), the Cmax of payload after multiple doses was much lower with only 1.48 ng/mL was detected. All together, these data indicated a good preclinical safety profile of SHR-A1811 and potential fewer toxicity during treatment.

**Table 3:** GLP toxicology study summary of SHR-A1811 in cynomolgus monkeys.

Doses	3 mg/kg	10 mg/kg	40 mg/kg
No. of animal	5/sex/group		
Regimens	Intravenous, every 3 weeks (5 times in total)		
Mortality	No report	No report	No report
Clinical signs	Normal	Normal	Normal
Body weight and food intake	Normal	Normal	Normal
Cardiac function	Normal	Normal	Normal
Hematology	Normal	Normal	Reticulocyte number and percentage decrease by 80%
Clinical chemistry at day2	Normal	Normal	Normal
Target organs and tissue	Normal	Normal	Thymus weight and organ coefficient decrease by 71%
HNSTD	40 mg/kg		

**Table 4:** Toxicokinetics parameters of SHR-A1811 (40 mg/kg) in cynomolgus monkeys after single and multiple doses.

TK Parameters	Payload		Total antibody		SHR-A1811	
Single	Multiple	Single	Multiple	Single	Multiple
Tmax (day)	0.269	0.350	0.0569	0.0243	0.0406	0.0243
Cmax (ng/ml)	1.44	1.48	779038	1046468	649048	801109
AUC_t_ (day*ng/ml)	5.76	6.34	4232223	5451542	3111089	3988273
AUC_INF_ (day*ng/ml)	6.67	7.14	4904112	6316239	3489237	4524900
t1/2 (day)	4.46	4.21	7.53	7.49	6.80	7.06
RacAUC_INF_	/	1.07	/	1.29	/	1.30

## Discussion

The adverse effects of ADCs result from both on-target and off-target toxicities, the latter often caused by systemic release of free payloads and non-specific uptake of antibodies. The mechanisms underlying why T-DXd induced ILD/pneumonitis remain unclear. Considering the adequate blood flow and long retention time in lung, the free payload in circulation may lead to lung damage [25]. There is also a low expression level of HER2 on pulmonary bronchial epithelial cells. Recent studies in monkeys described that target-independent uptake of T-DXd into alveolar macrophages might be another possibility [26]. The success of the 3^rd^-generation ADC has proven that the bystander killing effect can overcome the challenges such as tumor penetration and antigen heterogeneity, and dramatically improve the clinical benefits in solid tumors [10,27]. However, despite a stable linker and high systemic clearance of payload were designed to minimize toxicities, the high rate of associated adverse effects were frequently reported and even lead to treatment discontinuation. Therefore, to reduce the undesired toxicities of anti-HER2 ADC, we aim to develop a novel SHR-A1811 with optimal cytotoxicity, high stability, and potential less toxicity.

To achieve this purpose, SHR169265 was designed via introducing a chiral cyclopropyl at the carbonyl alpha position of exatecan derivative (Fig 1A). Such structural modification improved lipophilicity, membrane permeability and systemic clearance. The inhibitory potency on topoisomerase I was retained (Table 1 and S1 Fig). The faster clearance could decrease the systemic exposure to free payload and associated toxicities. The increased membrane permeability eventually led to the strong cytotoxicity of SHR169265 (Table 2), and optimal bystander killing capacity of ADCs. SHR-A1811 with DAR 6 demonstrated a comparable bystander killing potency with HRA18-C015 (a synthesized T-DXd, DAR 8) (Figs 1B and C), although the *in vitro* cytotoxicity of SHR-A1811 was slightly weaker (Table 2). SHR-A1811 treatment caused a potent and sustained inhibition of tumor growth in a dose-dependent manner, comparable with HRA18-C015, across HER2 low-to-high expressing xenograft models (Fig 2). The tumoral concentration of payload was proportional to the dosages of ADCs, while circulating payload was undetectable. Payload accumulation in tumors may contribute to the potent antitumor activity of SHR-A1811. Taken together, SHR-A1811 with DAR 6 showed strong enough antitumor efficacy although with less DAR compared to HRA18-C015 (DAR 8).

The chemical modification of SHR169265 was anticipated to form a proper steric hindrance and shield when connecting with the linker, and thus enhance the chemical stability of linker-payload system and conjugated SHR-A1811. As expected, SHR-A1811 showed high stability in plasma of different species with only less than 1% of payload was released after 21-day incubation (Fig 3B). The reported release rate of payload from T-DXd ranged from 1.2% to 3.9% in mouse, rat, monkey, and human plasma [8]. Consistent with the *in vitro* observation, SHR-A1811 had similar PK profile with total antibody following single intravenous injection in rats (Fig 3A).

The high stability of SHR-A1811 was further confirmed by the toxicokinetics study in cynomolgus monkey. Concentrations of SHR-A1811 and total antibody in plasma after multiple doses were similar. In contrast to the high maximum concentration detected with SHR-A1811, the maximum concentration of payload was only approximately one part of 500,000 of SHR-A1811 (801109 *vs.* 1.48 ng/mL) (Fig 4 and table 4). The above results suggested that SHR-A1811 has high stability and potentially low toxicity.

The preclinical toxicology studies of T-DXd in cynomolgus monkeys found that the HNSTD of T-DXd was 30 mg/kg with administration once every 3 weeks for 3 doses. The major target organs were bone marrow, pulmonary, intestine, skin and testis [8]. Consistently, the most common adverse reactions of T-DXd in patients were neutropenia, leukopenia and anemia, *etc.*, with ILD/pneumonitis as AE of special interest [12]. In our hands, administration of 40 mg/kg SHR-A1811 in monkeys once every 3 weeks for up to 4 months (total 5 doses) led to decrease in reticulocyte number and thymus weight, without observations in other organs. The HNSTD of SHR-A1811 in monkeys was determined as 40 mg/kg (Table 3). The favorable safety profiles possibly could be well explained by the lower drug load, trace amount of free toxin in circulation and high systemic clearance of payload. Collectively, SHR-A1811 is a novel anti-HER2 ADC that has great tumor killing potency, high stability, and low toxicity with the best-in-class potential.

In the multi-center, dose-escalation phase I clinical trial (NCT04446260), SHR-A1811 was well tolerated and showed promising antitumor activity in heavily pretreated patients with HER2-expressing or mutant advanced cancers. Patients received intravenous doses of SHR-A1811 ranging from 1 to 8.0 mg/kg every 3 weeks with median duration of follow up at 8.9 months. The ORR in HER2-positive and HER2 low-expressing breast cancer patients were 76.3% and 60.4%, respectively [28]. The ORR in HER2-expressing/mutant non-breast solid tumor patients, including biliary tract cancers, colorectal cancer, gastric cancer, gastroesophageal junction adenocarcinoma and colorectal cancer, was 45.9%. The incidence of all grade ILD was 2.6%. It was worth noting that, of the 113 patients in the 6.4 mg/kg group, only 1 patient had drug-related ILD [28]. In this dose cohort, the C_max_ and AUC_inf_ of free payload in plasma was 3.6 ug/mL and 26.7 ug·day/mL after multiple dosing [28]. Notably, the reported ILD incidence of T-DXd at the same dosage in breast cancer patients was 21% [16] with the C_max_ and AUC _inf_ of free payload at 6.8 ug/mL and 34.2 ug·day/mL [29]. We speculated that the less DAR, low free payload plasma exposure, and optimal systemic clearance might contribute to the better tolerability of SHR-A1811 compared to T-Dxd. More studies are being conducted to validate the potency and safety of SHR-A1811.

In summary, with a highly permeable payload, optimized DAR, great potency and better safety profiles, SHR-A1811 has demonstrated the best-in-class potential. Currently SHR-A1811 has entered phase II and phase III clinical studies for breast cancer, gastric cancer, colorectal cancer, and NSCLC (NCT05424835, NCT05482568, NCT04818333, NCT05349409).

## Supporting information

S1 Fig The inhibitory effect of SHR169265 and SHR167971 on topoisomerase I activity. (TIF)

S2 Fig The relative mean fluorescence intensity (MFI) of cells by FACS analysis of HER2 expression. (TIF)

S3 Fig Anti-HER2-SHR169265 ADCs induced histone H2A. **X phosphorylation, cleaved PARP (A, B), and caspase 3/7 activation (C) in SK-BR-3 cells.** (A) Western blots of phosphorylated H2A.X and cleaved PARP. The SDS-PAGE represented one of three independent experiments. (B) Quantification of Western blots of three independent experiments. *P < 0.05 versus control IgG-ADC. (C)*P < 0.05 versus HRA18-C015. Western blots in A and B were spliced for corresponding antibody incubations.
(TIF)

S4 Fig Body weight changes in NCI-N87 (A), JIMT-1 (B) and Capan-1 (C) xenograft models after SHR-A1811 treatment. (TIF)

S1 Table PK parameters of SHR-A1811 and HRA18-C015 in mice following 3 mg/kg intraperitoneal *vs.* intravenous administration. (DOCX)
