# Supplementary figures and images for: SHR-A1811, a novel anti-HER2 antibody–drug conjugate with optimal drug-to-antibody ratio, efficient tumor killing potency, and favorable safety profiles
Source: PLoS One. 2025 Jun 26;20(6):e0326691. doi: 10.1371/journal.pone.0326691 (PMC12200682; doi:10.1371/journal.pone.0326691)

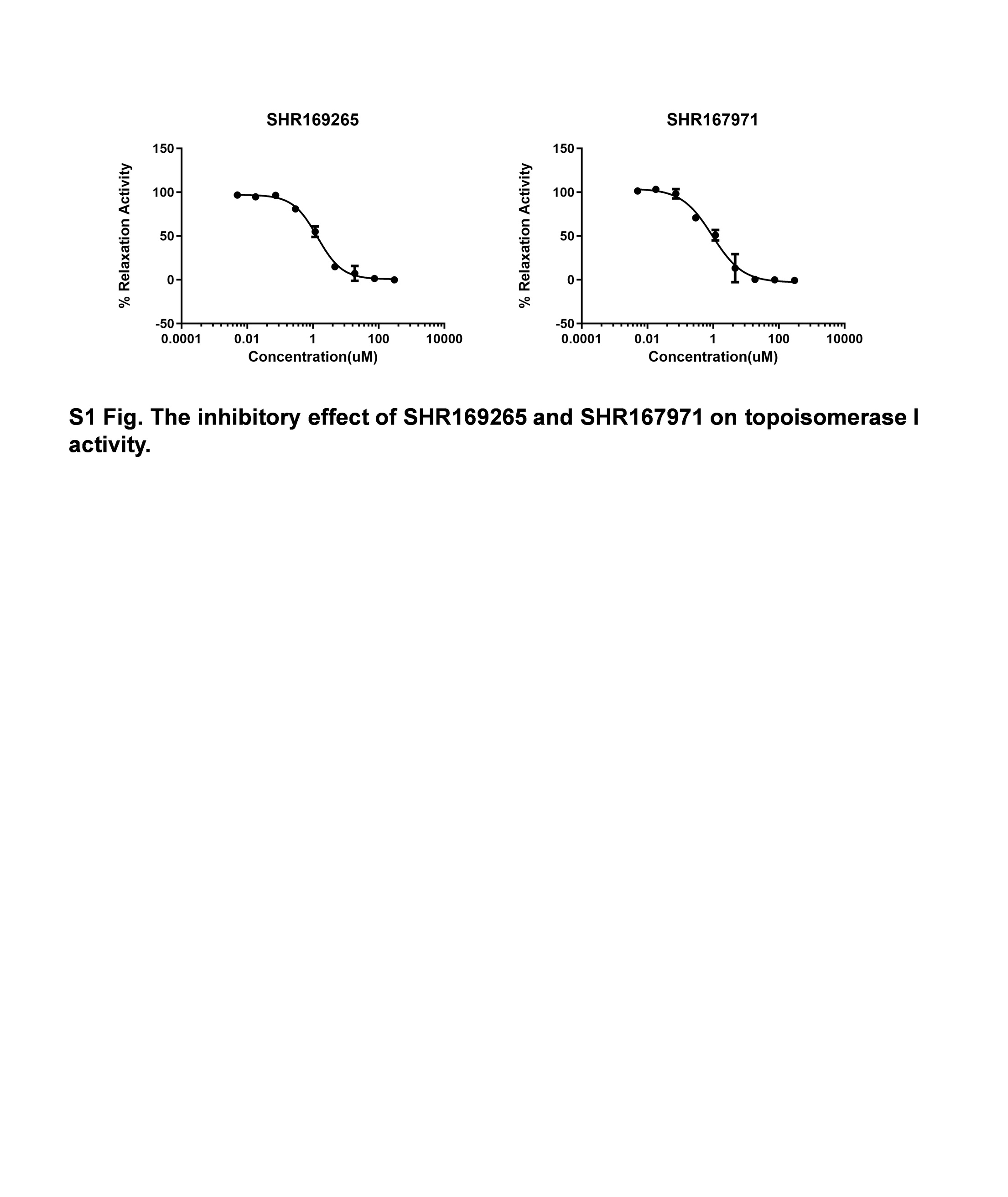

Supplement: S1 Fig — (TIF) [file pone.0326691.s001.tif]

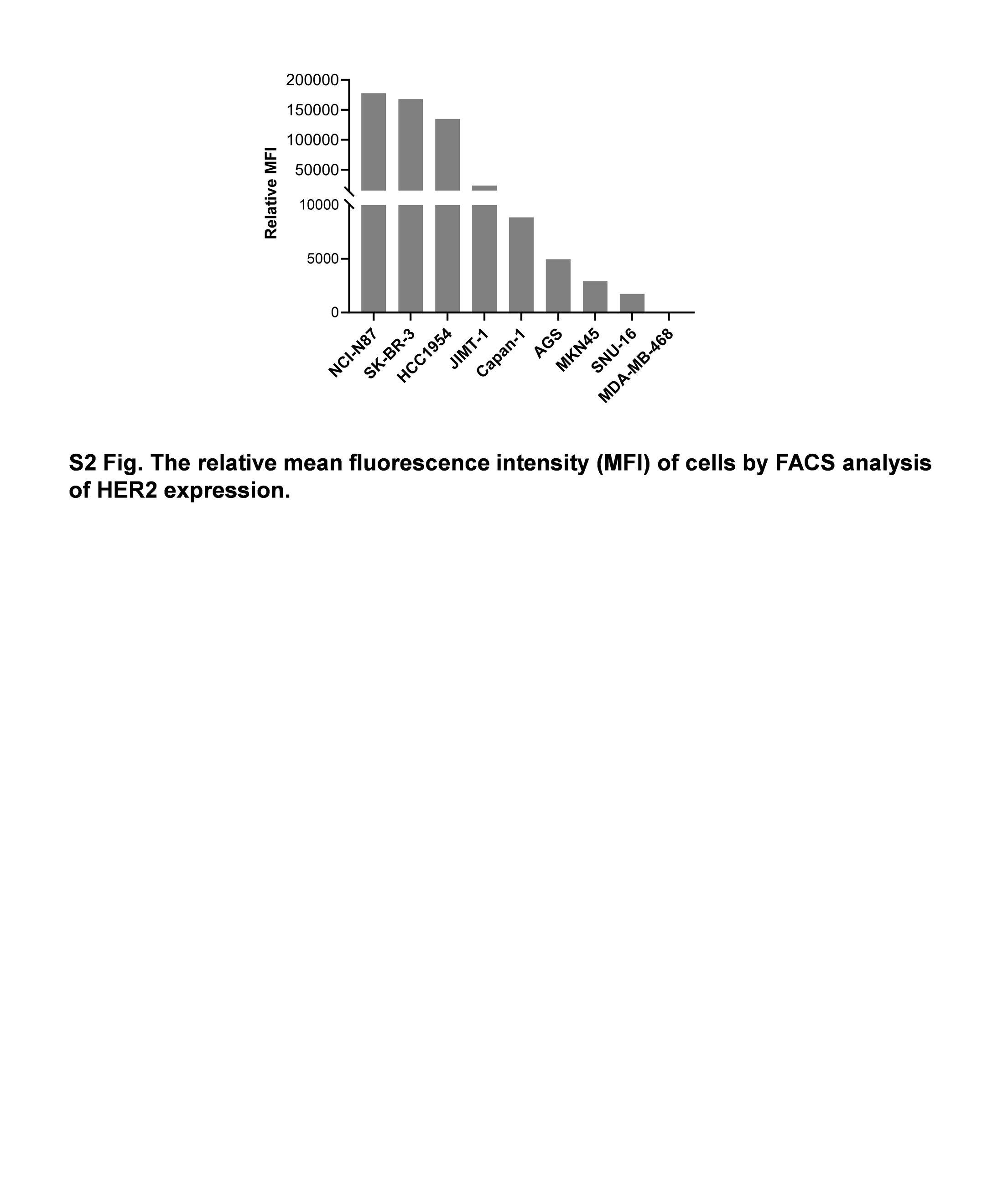

Supplement: S2 Fig — (TIF) [file pone.0326691.s002.tif]

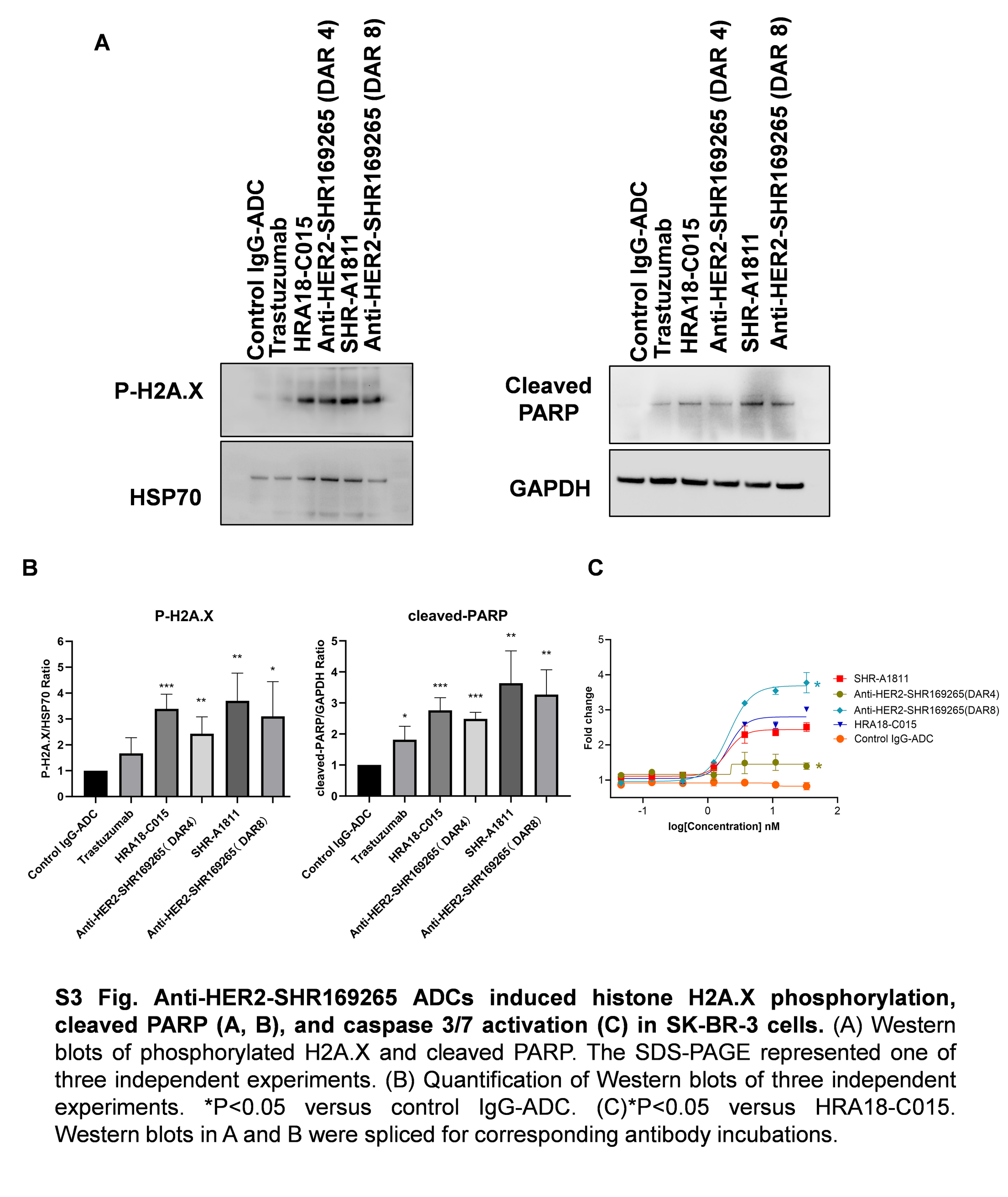

Supplement: S3 Fig — X phosphorylation, cleaved PARP (A, B), and caspase 3/7 activation (C) in SK-BR-3 cells. (A) Western blots of phosphorylated H2A.X and cleaved PARP. The SDS-PAGE represented one of three independent experiments. (B) Quantification of Western blots of three independent experiments. *P < 0.05 versus control IgG-ADC. (C)*P < 0.05 versus HRA18-C015. Western blots in A and B were spliced for corresponding antibody incubations. (TIF) [file pone.0326691.s003.tif]

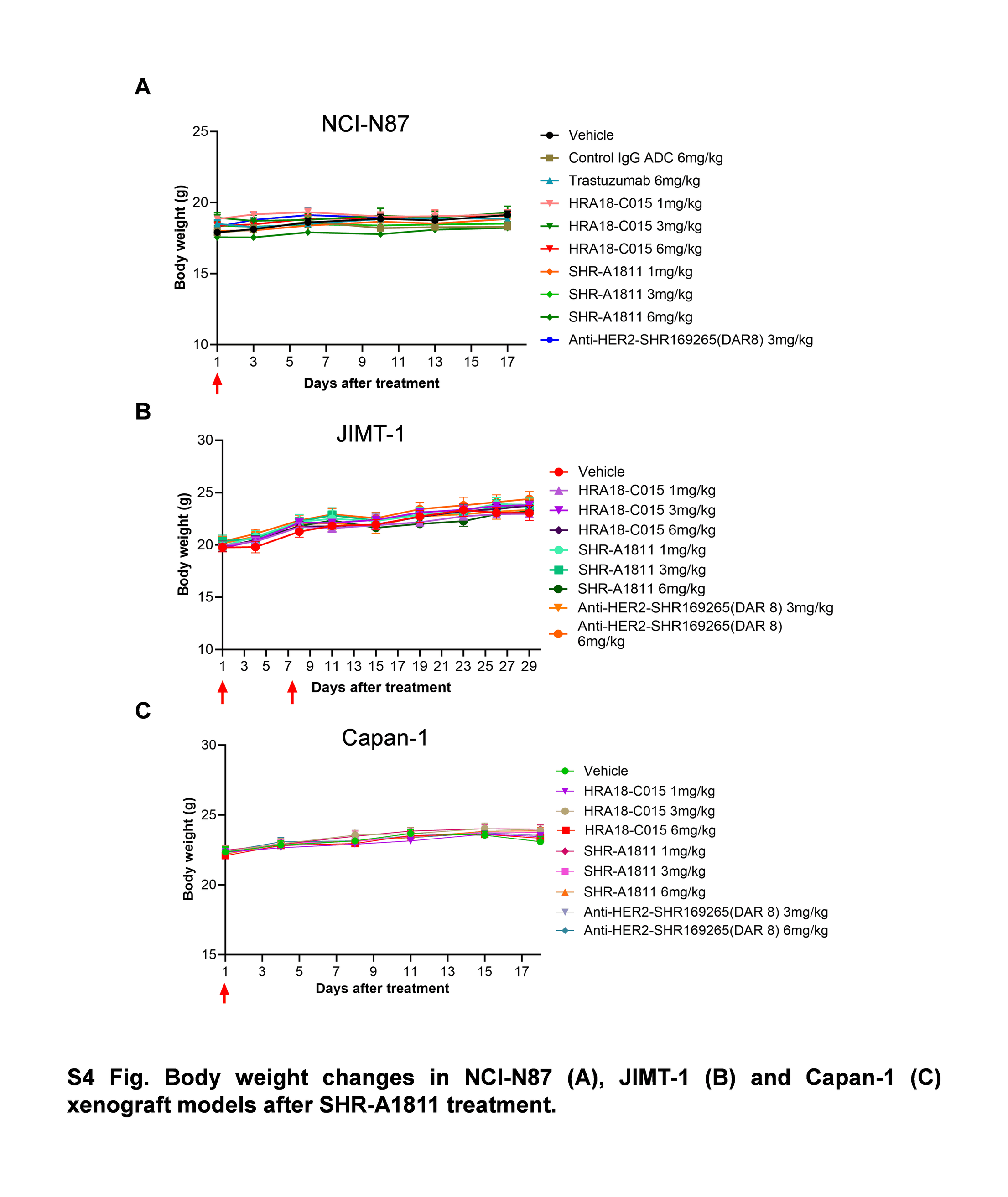

Supplement: S4 Fig — (TIF) [file pone.0326691.s004.tif]
