# Supplementary material for: SHR-A1811, a novel anti-HER2 antibody–drug conjugate with optimal drug-to-antibody ratio, efficient tumor killing potency, and favorable safety profiles
Source: PLoS One. 2025 Jun 26;20(6):e0326691. doi: 10.1371/journal.pone.0326691 (PMC12200682; doi:10.1371/journal.pone.0326691)
Supplement: S1 Table — (DOCX) [file pone.0326691.s005.docx]

S1 Table: PK parameters of SHR-A1811 and HRA18-C015 in mice following 3mg/kg intraperitoneal *vs.* intravenous administration

| PK Parameters | SHR-A1811  IV @ 3mpk | SHR-A1811  IP @ 3mpk | HRA18-C015  IV @ 3mpk | HRA18-C015  IP @ 3mpk |
| --- | --- | --- | --- | --- |
| t_1/2_ (d) | 4.7 ± 1.4 | 4.6 ± 1.6 | 4.5±1.2 | 5.8±1.7 |
| Cmax (μg/mL) | 59.6±12.2 | 18.1±3.1 | 68.6±3.4 | 21.4±2.3 |
| AUC _0-672_ (μg/mL*h) | 2054.6±207.8 | 2037.9±858.0 | 2992.2±1297.9 | 3062.8±656.2 |
| AUC _0-∞_ (μg/mL*h) | 2087.3±233.4 | 2080.7±894.5 | 3044.2±1308.6 | 3186.3±744.6 |
| Vz (mL/kg) | 234.9±61.1 | 241.5±43.8 | 160.2±28.4 | 189.9±48.9 |
| CL (mL/day/kg) | 34.8±3.9 | 42.6± 25.5 | 27.1±10.9 | 23.4±4.6 |
| MRT _0-∞_ (h) | 86.7 ± 38.2 | 132.8 ± 54.8 | 106.3±50.9 | 172.8±52.8 |
